# Prolonged versus intermittent β-lactam antibiotics intravenous infusion strategy in sepsis or septic shock patients: a systematic review with meta-analysis and trial sequential analysis of randomized trials

**DOI:** 10.1186/s40560-020-00490-z

**Published:** 2020-10-06

**Authors:** Yutaka Kondo, Kohei Ota, Haruki Imura, Naoki Hara, Nobuaki Shime

**Affiliations:** 1 Department of Emergency and Critical Care Medicine, Juntendo University Urayasu Hospital, 2-1-1 Tomioka, Urayasu, Chiba, 279-0021 Japan; 2 Department of Emergency and Critical Care Medicine, Graduate School of Biomedical & Health Sciences, Hiroshima University, Kasumi 1-2-3, Minami-ku, Hiroshima, 734-8551 Japan; 3 Department of Infectious Diseases, Rakuwakai Otowa Hospital, Otowachinjicho 2, Kyoto-shi, Yamashina-ku, Kyoto, 607-8062 Japan; 4 Japan Organization of Occupational Health and Safety, Yokohama Rosai Hospital, 3211 Kozukue, Kohoku, Yokohama, Kanagawa 222-0036 Japan

**Keywords:** Antibiotics, Pharmacokinetic, Continuous infusion, Sepsis, Septic shock

## Abstract

**Background:**

The prolonged β-lactam infusion strategy has emerged as the standard treatment for sepsis or septic shock despite its unknown efficacy. This study aimed to assess the efficacy of prolonged versus intermittent β-lactam antibiotics infusion on outcomes in sepsis or septic shock patients by conducting a systematic review and meta-analysis.

**Methods:**

A thorough search was conducted on MEDLINE, the Cochrane Central Register of Controlled Trials, and the Igaku Chuo Zasshi databases. Randomized controlled trials (RCTs) comparing mortality between prolonged and intermittent infusion in adult patients with sepsis or septic shock were included. The primary outcome was hospital mortality. The secondary outcomes were the attainment of the target plasma concentration, clinical cure, adverse events, and occurrence of antibiotic-resistant bacteria. We performed a subgroup analysis stratified according to the year of publication before or after 2015 and a trial sequential analysis (TSA). The Der Simonian–Laird random-effects models were subsequently used to report the pooled risk ratios (RR) with confidence intervals (CI).

**Results:**

We identified 2869 studies from the 3 databases, and 13 studies were included in the meta-analysis. Hospital mortality did not decrease (RR 0.69 [95%CI 0.47–1.02]) in the prolonged infusion group. The attainment of the target plasma concentration and clinical cure significantly improved (RR 0.40 [95%CI 0.21–0.75] and RR 0.84 [95%CI 0.73–0.97], respectively) in the prolonged infusion group. There were, however, no significant differences in the adverse events and the occurrence of antibiotic-resistant bacteria between the groups (RR 1.01 (95%CI 0.95–1.06) and RR 0.53 [95%CI 0.10–2.83], respectively). For the subgroup analysis, a significant improvement in hospital mortality or clinical cure was reported in studies published in or after 2015 (RR 0.66 [95%CI 0.44–0.98] and RR 0.67 [95%CI 0.50–0.90], respectively). The results of the TSA indicated an insufficient number of studies for a definitive analysis.

**Conclusions:**

The prolonged infusion of β-lactam antibiotics significantly improved upon attaining the target plasma concentration and clinical cure without increasing the adverse event or the occurrence of antibiotic-resistant bacteria. Prolonged infusion could not improve hospital mortality although an improvement was shown for studies published in or after 2015. Further studies are warranted as suggested by our TSA results.

## Introduction

Sepsis and septic shock can cause high morbidity and mortality rates; thus, the early and appropriate use of effective antibiotics is important [1]. β-Lactam antibiotics are antibiotics commonly used by sepsis or septic shock patients in intensive care units (ICU) [2]. Traditionally, β-lactam antibiotics have been administered via intermittent intravenous infusion. However, there remain doubts regarding the intermittent infusion strategy [3]. This is because the maintenance of concentrations above the minimum inhibitory concentration (MIC) of pathogens is associated with bacterial clearance [4]. The prolonged infusion of β-lactams can maintain the plasma concentrations of antibiotics above the MIC, which may improve clinical outcomes, hence the emergence of the prolonged β-lactam antibiotics infusion strategy.

The current international guidelines on the management of sepsis and septic shock (Surviving Sepsis Campaign, 2016) recommended that dosing strategies of antimicrobials be optimized based on the accepted pharmacokinetic/pharmacodynamics principles and the specific drug properties [5]. However, this has not been clearly defined for the prolonged β-lactam antibiotics infusion strategy.

Several systematic reviews were conducted to evaluate the utility of the prolonged infusion of β-lactam antibiotics [6–8]. A systematic review published in 2011 could not show that prolonged β-lactam infusion significantly improved clinical outcomes [6]. No international guidelines have suggested the use of β-lactam antibiotics administered via continuous infusion or extended infusion in treating sepsis and septic shock [7]. However, some recent studies have revealed that the prolonged infusion of β-lactam antibiotics significantly improved hospital mortality [8, 9], adding to the controversy surrounding the efficacy of the prolonged β-lactam infusion strategy. The efficacy of a prolonged β-lactam infusion strategy may change over time.

This study aimed to conduct a systematic review and meta-analysis of the present randomized controlled trials (RCTs) to assess the efficacy of the prolonged versus intermittent β-lactam antibiotics infusion strategy on outcomes in sepsis or septic shock patients.

## Material and methods

### Data sources and search strategies

To identify eligible trials, we searched the MEDLINE (via PubMed), Cochrane Central Register of Controlled Trials, and Igaku Chuo Zasshi (ICHUSHI; Japanese) databases. Searches were not restricted by publication status, date of publication, or sample size. Studies published in English or Japanese were included. We searched for articles on April 27, 2019; the search strategies are presented in Additional file 1. The systematic review and meta-analysis were conducted as per the PRISMA guidelines [10] and were registered in the UMIN Clinical Trials Registry (ID: UMIN000040688).

### Study selection

The titles and abstracts of the search results were retrieved from the aforementioned databases. After excluding duplicated studies, two reviewers (KO and HI) independently screened the titles and abstracts of the studies for potential eligibility. A third reviewer (YK) was consulted when the two independent reviewers disagreed. If disagreement persisted, the full text of the paper was obtained to determine the eligibility of the study. The full texts of articles included in the final selection were independently reviewed by KO and HI, and eligible studies were consulted on by a third reviewer (YK), and resolution of discrepancies was determined after discussion.

Studies were identified in accordance with the research question formulated based on the participants, interventions, comparisons, and outcome models: P, adult (≥ 18 years of age) patients diagnosed with sepsis or septic shock admitted to the ICU; I, a prolonged β-lactam antibiotics infusion strategy (continuous or extended time [greater than 1 h but not continuous] of intravenous infusion); C, an intermittent β-lactam antibiotics infusion strategy (within 1 h of intravenous infusion); and O, all-cause mortality. The definitions regarding sepsis were not restricted to the latest definition (Sepsis-3) [11]; instead, past sepsis definitions were allowed for the included studies.

### Data extraction

Data extraction was conducted independently by two investigators (KO and HI). The data extracted included authors, year of publication, country, study design, number and type of participants, the severity of the patient’s diagnosis, inclusion period, outcome measures, and study results.

### Study endpoints

The hospital mortality was set as the primary outcome. The secondary outcomes were the attainment of the target plasma concentration, clinical cure, adverse event, and the occurrence of antibiotic-resistant bacteria.

### Subgroup analysis

For the subgroup analysis, we stratified studies by the published year into before and after 2015. For this analysis, the outcome was hospital mortality and clinical cure. Subgroup analysis was not pre-specified before the systematic review process.

### Assessment of methodological quality: risk of bias assessment and GRADE approach

We adapted the Cochrane risk of bias tool to assess the quality of the studies included in the meta-analysis [12]. Each study was assessed for (i) random sequence generation (selection bias), (ii) allocation concealment (selection bias), (iii) blinding of participants and staff (performance bias), (iv) blinding of related outcome assessments (detection bias), (v) true intention-to-treat analysis (attrition bias), (vi) incomplete outcome data (attribution bias), (vii) selective reporting (reporting bias), (viii) early trial withdrawal bias, and (ix) other sources of bias. Two investigators (KO and HI) independently assessed the risk of bias of the included studies and classified the studies as having a low, intermediate, or high risk of bias in each domain. If discrepancies emerged, it was resolved by a third investigator (YK) via an independent evaluation.

We graded the quality of evidence of each finding based on the criteria established by the Grading of Recommendations Assessment, Development, and Evaluation (GRADE) working group [13]. The quality of the study methodology was classified as high, intermediate, low, or very low, and it was based on the study design, risk of bias, indirectness, inconsistency, imprecision, and publication bias. The publication biases were assessed visually by inspecting the funnel plots.

### Statistical analysis

We pooled the eligible patients for each outcome and calculated the risk ratios (RR) and corresponding 95% confidence intervals (CI) using the Der Simonian–Laird random-effects model with weights calculated by the Mantel–Haenszel method [14]. We verified the heterogeneity of the included studies using the estimated Cochrane chi-square test, Tau^2^, and the *I*^2^ statistics (*I*^2^ > 50% indicated severe heterogeneity). We applied the unadjusted *p* values to assess the significance, with cutoffs for two-tailed *p* values of 0.05 for hypothesis testing and 0.1 for heterogeneity testing. All statistical analyses were performed using Review Manager, Cochrane systematic review software, version 5.3.5 for Windows (The Nordic Cochrane Centre, the Cochrane Collaboration, Copenhagen, Denmark).

### Trial sequential analysis

We applied trial sequential analysis (TSA) to the meta-analysis to search for the possibility of false-positive (type I error) or false-negative (type II error) results [15]. We set the overall two-sided type I error at 5% and set 80% power to calculate the diversity-adjusted information size for the analysis. An anticipated relative risk reduction (RRR) of hospital mortality was set at 30% between the groups. TSA viewer version 0.9.5.10 Beta (Copenhagen Trial Unit, Centre for Clinical Intervention Research, Copenhagen, Denmark, 2016) was used.

## Results

### Search results

**Fig. 1:**
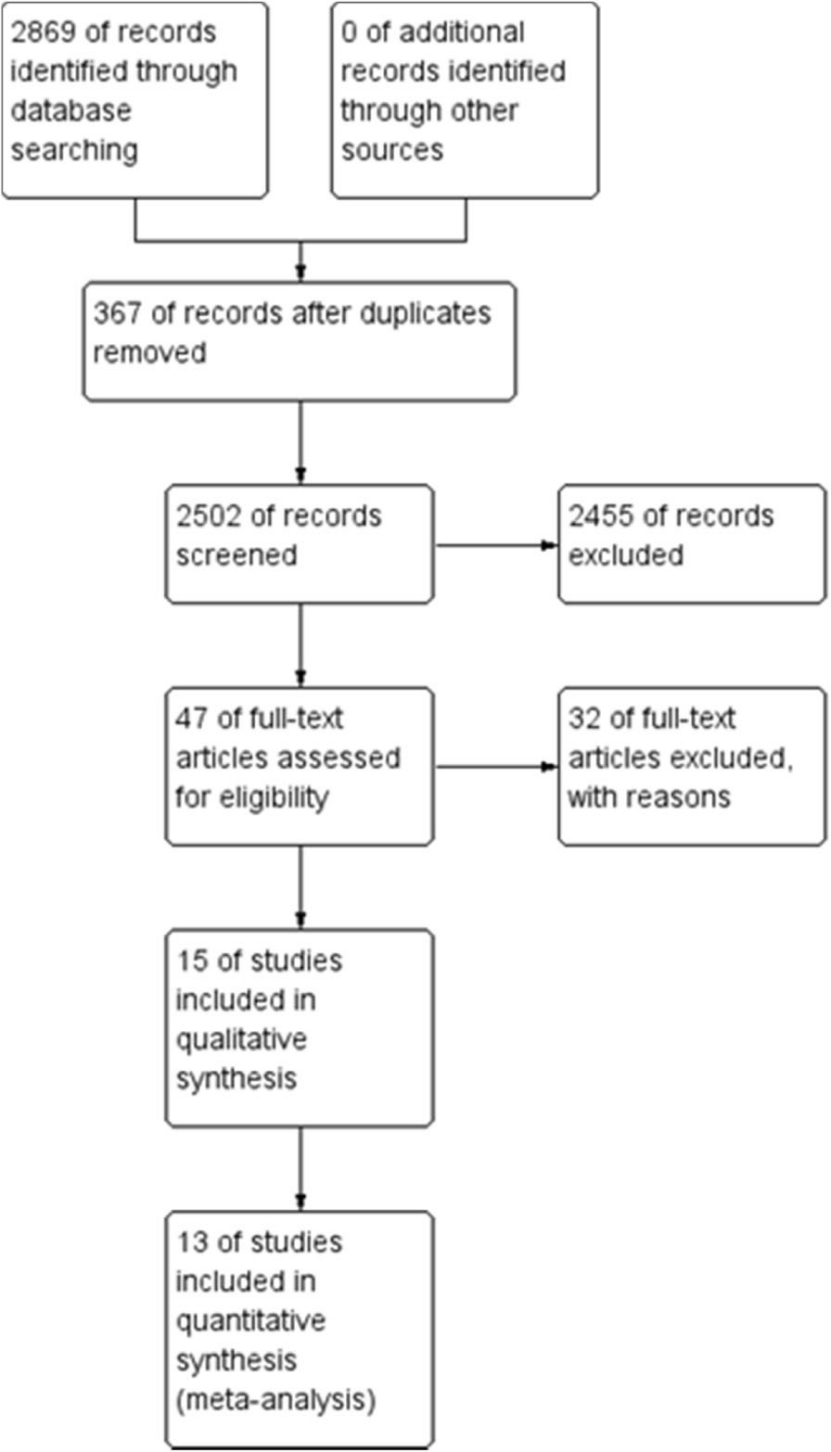
Flow diagram of the search strategy and study selection. MH, Mantel–Haenszel; CI, confidence interval

We identified 2869 studies from the electronic databases. After the elimination of duplicates, 47 studies were eligible based on the assessment of titles and abstracts. A further 32 studies were excluded based on the review of full-text articles as they reviewed or reported the same trials of other included publications despite having different study designs. Two studies were also excluded because they did not contain the outcomes for meta-analysis; therefore, 13 studies were included in the meta-analysis (Fig. 1).

### Study characteristics

The individual characteristics of trials included in the meta-analysis are detailed in Table 1 [16–28]. Four RCTs were performed in Asia [16, 17, 25, 28], three in Europe [18, 20, 21], two in North America [22, 23], two in Oceania [26, 27], and two in multiple countries [19, 24]. Regarding the identified organism, five RCTs [16, 17, 25, 27, 28] involved treatment for Gram-negative bacteria only, whereas the others included several kinds of bacteria. The dosing of antibiotics varied between the included studies. For the primary outcome, a total of 825 patients from nine RCTs [3, 17, 19–21, 24, 25, 27, 28] were included in the analysis of the hospital mortality, and 410 were assigned to the prolonged infusion group, while 415 were assigned to the intermittent infusion group. For the secondary outcomes, 177 patients from two RCTs [16, 19] were included in the attainment of the target plasma concentration, 886 patients from nine RCTs [16, 18–23, 26, 28] were included in clinical cure, 691 patients from three RCTs [21, 22, 24] were included in the adverse event groups, and 198 patients from one RCT [18] were included in the occurrence of antibiotic-resistant bacteria group.

**Table 1:** Characteristics of included studies

No.	First author, year	Country	Design	Double blinding	Number of study participants, total (P vs I)	Age, P vs I, median	APACHEII, P vs I, median	Sepsis definition or type of infection	Organism	Antibiotics	Prolonged infusion	Intermittent infusion
1	Angus, 2000	Thailand	RCT	NR	21 (10 vs 11)	43 vs 48	21 vs 15	Septic melioidosis	Only Gram-negative	β-Lactam (unknown in detail)	12 mg/kg loading dose, followed by 4 mg/kg/h by constant rate infusion	40 mg/kg every 8 h, intermittent bolus injection
2	Nicolau, 2001	USA	RCT	No	35 (17 vs 18)	46 vs 56 (mean)	13.9 vs 15.5 (mean)	Nosocomial pneumonia	Mostly Gram-negative	β-Lactam (ceftazidime) plus tobramycin	Ceftazidime, 3 g/day, continuously; tobramycin, 7 mg/kg/day	Ceftazidime: 2 g, over 30 min, every 8 h; tobramycin, 7 mg/kg/day
3	Georges, 2005	France	RCT	No	50 (26 vs 24)	50 vs 46 (mean)	^a^45 vs 44 (^a^SAPS II, median)	Nosocomial pneumonia or bacteremia	Mostly Gram-negative	β-Lactam (cefepime) plus amikacin	Cefepime, 4 g/day, continuously; amikacin, 20 mg/kg loading dose, followed by 15 mg/kg/day	Cefepime, 2 g, over 30 min, twice daily; amikacin: 20 mg/kg loading dose, followed by 15 mg/kg/day
4	Rafati, 2006	Iran	RCT	NR	40 (20 vs 20)	50.1 vs 48.0 (mean)	16.4 vs 14.2 (mean)	Septic patients with SIRS (Sepsis-2)	Only Gram-negative	β-Lactam (piperacillin) plus amikacin	Piperacillin, 2 g loading dose, followed by 8 g/day, continuously; amikacin, 15 mg/kg/day	Piperacillin, 3 g, over 30 min, every 6 h; amikacin, 15 mg/kg/day
5	Lau, 2006	USA	RCT	No	258 (128 vs 130)	51.5 vs 48.0	7 vs 7	Complicated intra-abdominal infections	Gram-negative and Gram-positive	β-Lactam (piperacillin–tazobactam)	2.25 g loading dose, followed by 13.5 g/day, continuously	3.375 g, over 30 min, every 6 h
6	Roberts, 2007	Australia	RCT	No	57 (29 vs 28)	41 vs 56	20 vs 17	Sepsis (SIRS criteria)	Mixed	β-Lactam (ceftriaxone)	Continuous infusion, 2 g/day	Intermittent bolus, 2 g every 24 h
7	Roberts and Kirkpatrick, 2009	Australia	RCT	No	10 (5 vs 5)	57 vs 55	^a^5 vs 3 (^a^SOFA, median)	Sepsis (SIRS criteria)	Only Gram-negative	β-Lactam (meropenem)	Continuous infusion, 3 g/day	Intermittent bolus, 1 g every 8 h
8	Chytra, 2012	Czech Republic	RCT	No	214 (106 vs 108)	44.9 vs 47.2 (mean)	21.4 vs 22.1 (mean)	Sepsis (Sepsis-2)	Mostly Gram-negative	β-Lactam (meropenem)	Continuous infusion, loading dose 2 g; 4 g every 24 h	30 min; 2 g every 8 h
9	Dulhunty, 2013	Australia, Hong Kong	RCT	Yes	60 (30 vs 30)	54 vs 60 (mean)	21 vs 23 (mean)	Severe sepsis (Sepsis-2)	Mixed	β-Lactam (piperacillin–tazobactam, ticarcillin–clavulanate, or meropenem)	Continuous infusion, clinician chosen from piperacillin–tazobactam, ticarcillin–clavulanate, or meropenem	Intermittent bolus, clinician chosen from piperacillin–tazobactam, ticarcillin–clavulanate, or meropenem
10	Dulhunty, 2015	Australia, New Zealand, Hong Kong	RCT	Yes	432 (212 vs 220)	64 vs 65	21 vs 20	Severe sepsis (Sepsis-2)	Mixed	β-Lactam (piperacillin–tazobactam, ticarcillin–clavulanate, or meropenem)	Continuous infusion clinician chosen from piperacillin–tazobactam, ticarcillin–clavulanate, or meropenem	Intermittent bolus clinician chosen from piperacillin–tazobactam, ticarcillin–clavulanate, or meropenem
11	Laterre, 2015	Belgium	RCT	NR	28 (14 vs 14)	68 vs 65	17 vs 16	Pulmonary or abdominal infection in ICU	Mostly Gram-negative	β-Lactam (temocillin)	Loading dose (2 g) administered over 30 min in 50 mL of water for injection followed by infusion (6 g in 48 mL of water for injection infused at a rate of 2 mL/h)	2 g of temocillin (in 50 mL of water for injection) every 8 h injected over a 30 min period
12	Abdul, 2016	Malaysia	RCT	No	140 (70 vs 70)	54vs 56	21 vs 21	Severe sepsis (Sepsis-2)	Mostly Gram-negative	β-Lactam (cefepime, meropenem or piperacillin/tazobactam)	^a^Continuous infusion	^b^Intermittent bolus
13	Zhao, 2017	China	RCT	No	50 (25 vs 25)	68 vs 67	19.4 vs 19.7	Severe sepsis and septic shock (Sepsis-2)	Only Gram-negative	β-Lactam (meropenem)	Loading dose of 0.5 g of meropenem followed by a continuous infusion of 3 g/day	Initial dose of 1.5 g followed by 1 g for every 8 h

*P* prolonged, *I* intermittent, *RCT* randomized control trial, *NR* not reported, *SAPS II* Simplified Acute Physiology Score II, *SOFA* Sequential Organ Failure Assessment, *SIRS* systemic inflammatory response syndrome, *ICU* intensive care unit

^a^Continuous infusion: Cefepime: day 1—2 g intravenous (IV) loading dose (infused over 30 min) followed by 2 g IV (infused over 480 min) every 8 h; day 2 onwards—2 g IV (infused over 480 min) every 8 h. Meropenem: day 1—1 g IV loading dose (infused over 30 min) followed by 1 g IV (infused over 480 min) every 8 h; day 2 onwards—1 g IV (infused over 480 min) every 8 h. Piperacillin/tazobactam: day 1—4 g/0.5 g IV loading dose (infused over 30 min) followed by 4 g/0.5 g IV (infused over 360 min) every 6 h; day 2 onwards—4 g/0.5 g IV (infused over 360 min) every 6 h

^b^Intermittent bolus: cefepime—2 g intravenous (IV) (infused over 30 min) every 8 h; meropenem—1 g IV (infused over 30 min) every 8 h; piperacillin/tazobactam—4 g/0.5 g IV (infused over 30 min) every 6 h

### Outcome

**Fig. 2:**
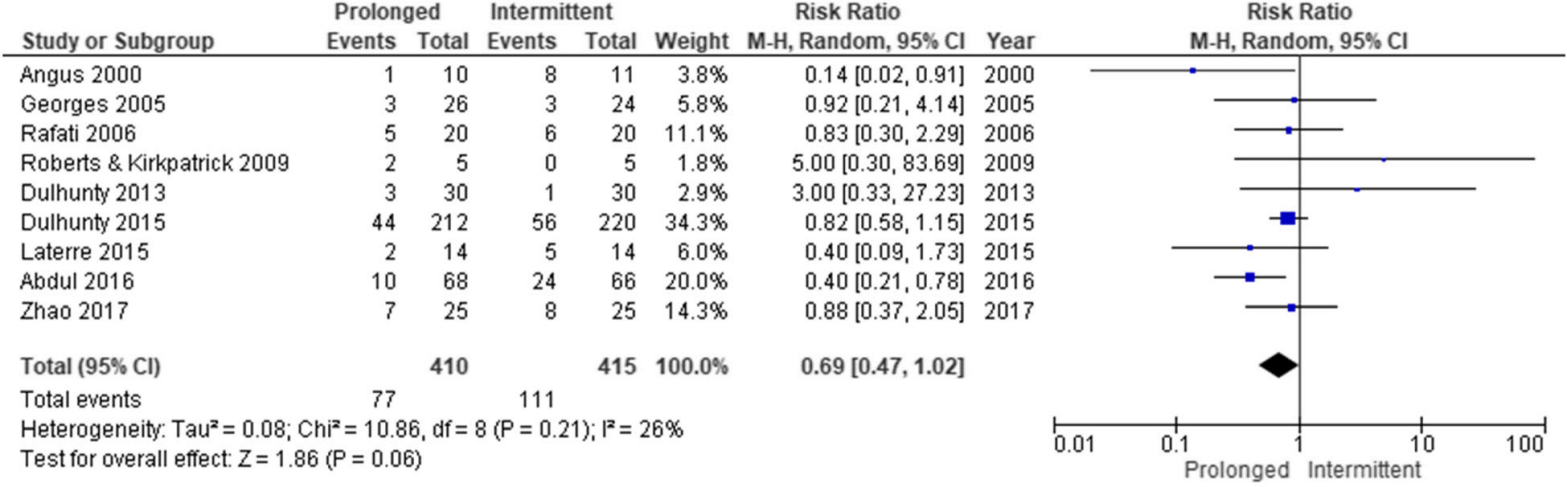
Forest plot comparing the hospital mortality between prolonged and intermittent infusion strategy in sepsis or septic shock patients. MH, Mantel–Haenszel; CI, confidence interval

**Fig. 3:**
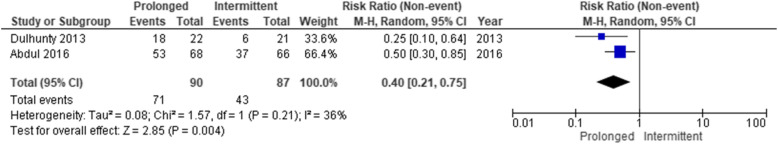
Forest plot comparing the attainment of the target plasma concentration between the prolonged and intermittent infusion strategy in sepsis or septic shock patients. MH, Mantel–Haenszel; CI, confidence interval

**Fig. 4:**
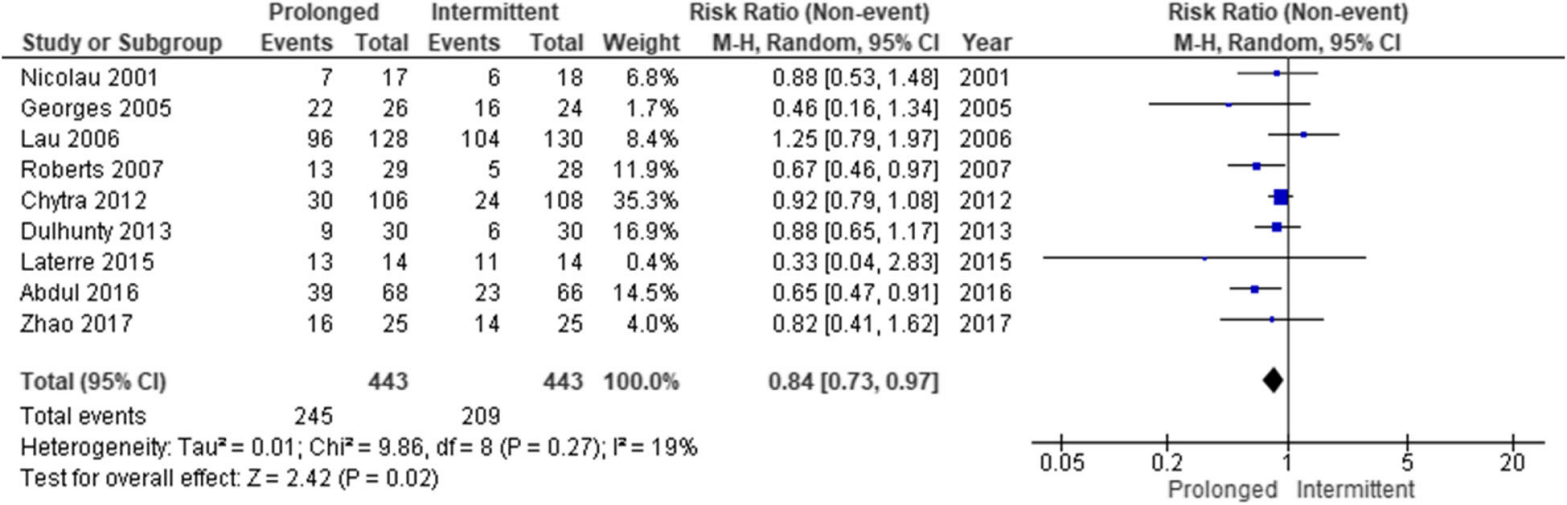
Forest plot comparing the clinical cure between prolonged and intermittent infusion strategy in sepsis or septic shock patients. MH, Mantel–Haenszel; CI, confidence interval

**Fig. 5:**
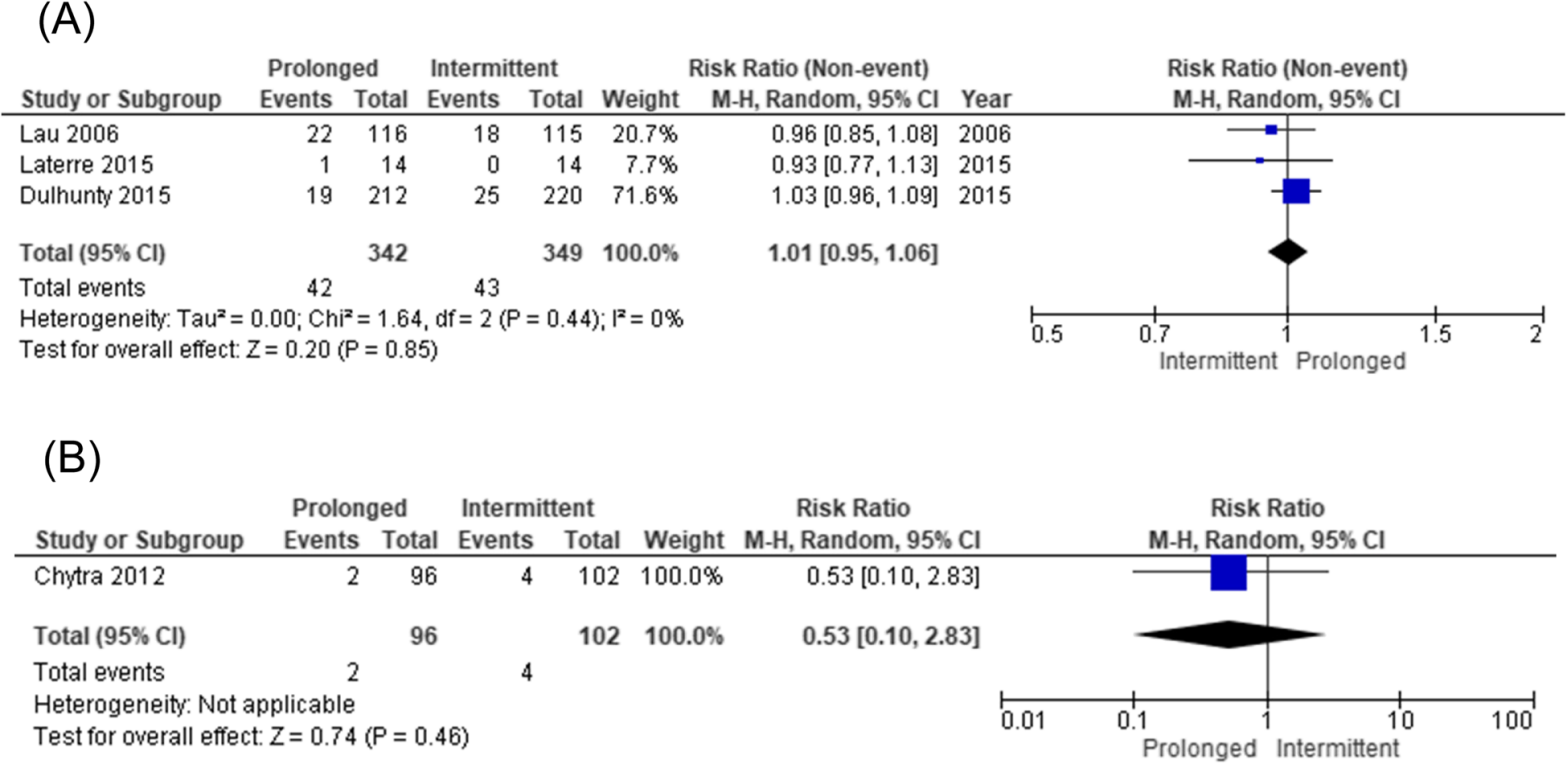
**a** Forest plot comparing the adverse events between prolonged and intermittent infusion strategy in sepsis or septic shock patients. **b** Forest plot comparing the occurrence of antibiotic-resistant bacteria between prolonged and intermittent infusion strategy in sepsis or septic shock patients. MH, Mantel–Haenszel; CI, confidence interval

The forest plot of the primary outcome is shown in Fig. 2. During hospitalization, 88 of the 420 patients (21.0%) died in the prolonged infusion group and 112 of 424 patients (26.4%) died in the intermittent infusion group. The pooled RR of hospital mortality did not decrease significantly (0.69 [95%CI 0.47–1.02]) in the prolonged infusion group. Regarding the secondary outcomes, the pooled RR of the attainment of the target plasma concentration and clinical cure significantly improved (0.40 [95%CI 0.21–0.75] and 0.84 [95%CI 0.73–0.97], respectively) in the prolonged infusion group (Figs. 3 and 4). There were no significant differences in the adverse event and the occurrence of antibiotic-resistant bacteria between the groups with pooled RR of 1.01 (95%CI 0.95–1.06) and 0.53 (95%CI 0.10–2.83), respectively (Fig. 5a, b).

For the subgroup analyses, studies of meta-analysis published before 2015 did not report an improvement in hospital mortality or clinical cure with pooled RR of 0.89 (95%CI 0.34–2.34) and 0.88 (95%CI 0.76–1.03), respectively. However, a significant improvement for hospital mortality or clinical cure was reported in studies published in or after 2015 with pooled RR of 0.66 (95%CI 0.44–0.98) and 0.67 (95%CI 0.50–0.90), respectively (Additional file 2).

### Heterogeneity

For the primary outcome of hospital mortality, heterogeneity among studies was not observed (*I*^2^ = 26.0%, *χ*^2^ = 10.86, *p* = 0.21) (Fig. 2). The evaluation of heterogeneity for secondary outcomes was described in each forest plot (Figs. 3, 4, and 5).

### Publication bias, risk of bias, and quality of evidence

**Fig. 6:**
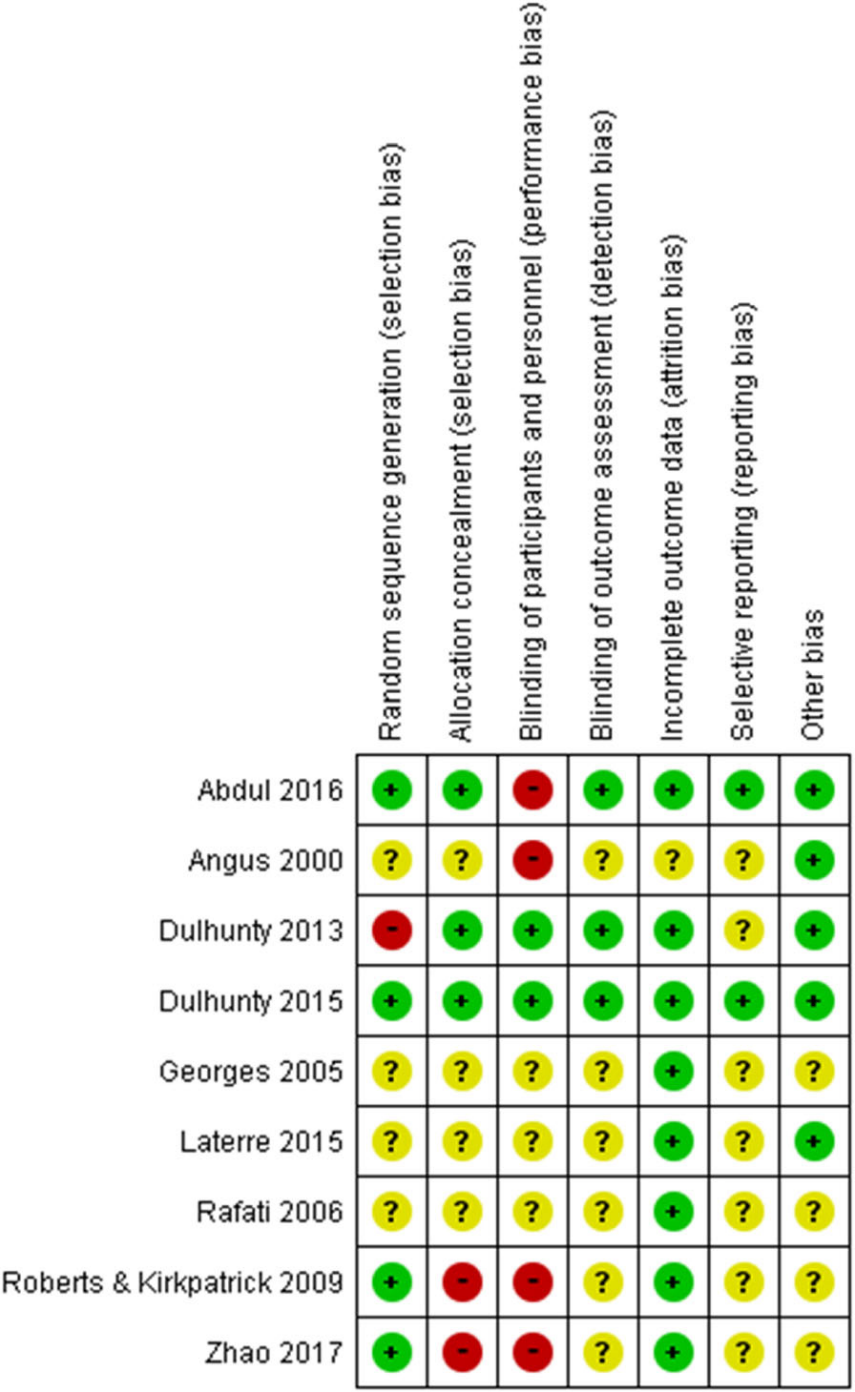
Risk of bias summary for the included studies

We tested for the presence of publication bias for the primary and secondary outcomes. A visual inspection of the funnel plot showed the absence of publication bias in hospital mortality (Additional file 3). As per the risk of bias for the primary outcome, blinding of participants and personnel was rated the highest (high risk of biases in 4 trials) (Fig. 6 and Additional file 4).

For the effect of prolonged versus intermittent infusion strategy on the primary outcome, the quality of evidence was rated as low; the grade was lowered by two points because of the risk of bias and imprecision of studies (such as the point estimate in each study being located in different directions in the funnel plot). The summary of the evidence for all outcomes is shown in Table 2.

**Table 2:** Summay table of findings

Outcomes	Anticipated absolute effects (95%CI)		Relative effect (95%CI)	Number of participants (studies)	Certainty of the evidence (GRADE)
	Risk with intermittent infusion	Risk with prolonged infusion			
Hospital mortality	267 per 1000	185 per 1000 (126 to 273)	RR 0.69 (0.47 to 1.02)	825 (9 RCTs)	⨁⨁◯◯ Low
Attainment of target plasma concentration	494 per 1000	198 per 1000 (104 to 371)	RR 0.40 (0.21 to 0.75)	177 (2 RCTs)	⨁⨁⨁◯ Moderate
Clinical cure	472 per 1000	396 per 1000 (344 to 458)	RR 0.84 (0.73 to 0.97)	886 (9 RCTs)	⨁⨁◯◯ Low
Adverse event	123 per 1000	124 per 1000 (117 to 131)	RR 1.01 (0.95 to 1.06)	691 (3 RCTs)	⨁⨁⨁◯ Moderate
Occurrence of antibiotic-resistant bacteria	39 per 1000	21 per 1000 (4 to 111)	RR 0.53 (0.10 to 2.83)	198 (1 RCT)	⨁⨁◯◯ Low

GRADE Working Group grades of evidence: High certainty—we are very confident that the true effect lies close to that of the estimate of the effect. Moderate certainty—we are moderately confident in the effect estimate: the true effect is likely to be close to the estimate of the effect, but there is a possibility that it is substantially different. Low certainty: our confidence in the effect estimate is limited: the true effect may be substantially different from the estimate of the effect. Very low certainty—we have very little confidence in the effect estimate: the true effect is likely to be substantially different from the estimate of the effect

*CI* confidence interval; *GRADE* Grading of Recommendations Assessment, Development, and Evaluation; *RR* risk ratio; *RCT* randomized controlled trial

### Trial sequential analysis

**Fig. 7:**
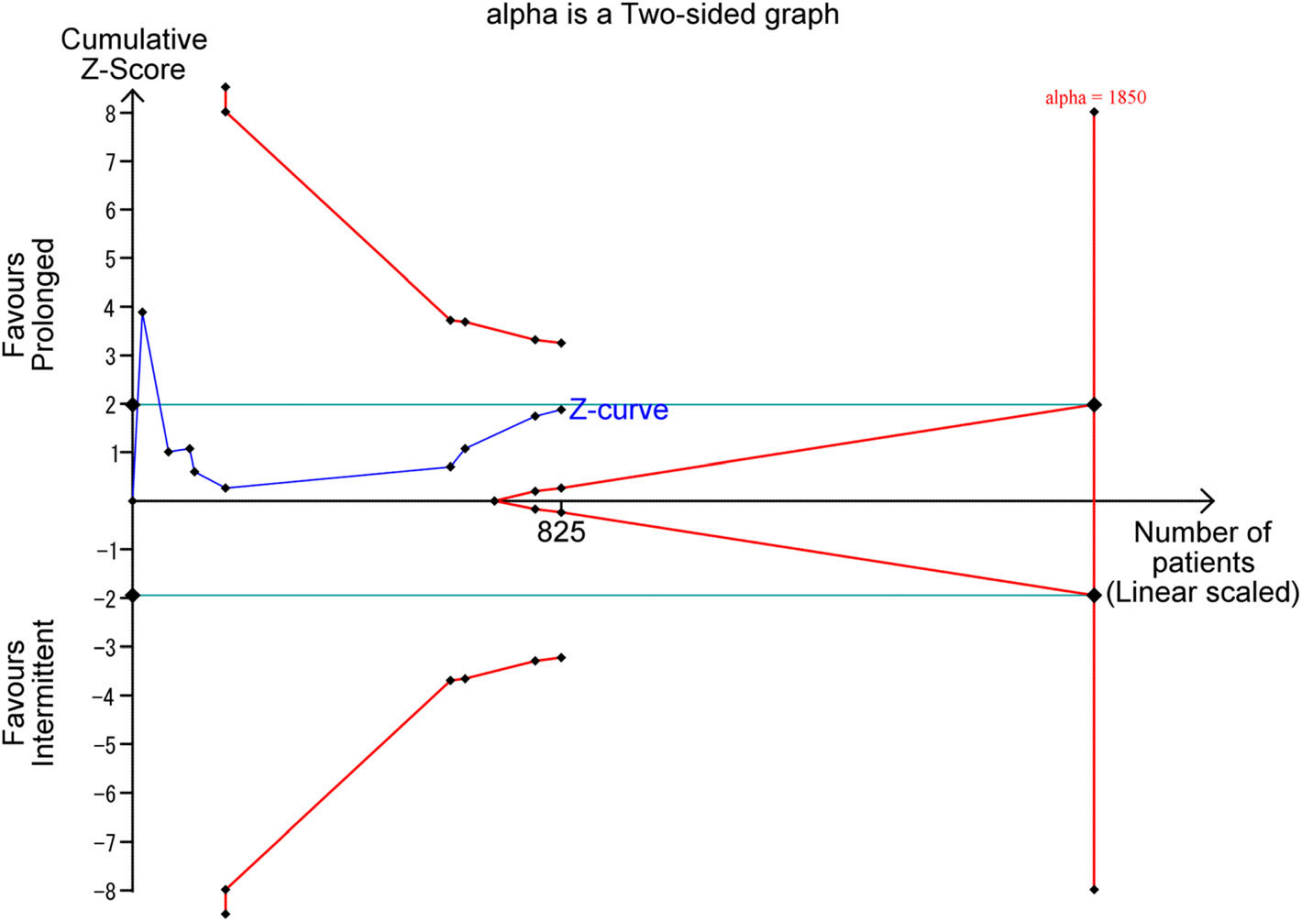
Trial sequential analysis for hospital mortality. Sample size: the diversity-adjusted information size; blue line, the cumulative *Z*-line; green line, the alpha boundary of significance; concaved red line, the TSA boundary

TSA showed the adjusted CI for hospital mortality was 0.37–1.30 (*I*^2^ = 26%; *n* = 825). The required information size to show a RRR of 30% was 1850. The cumulative *Z*-curve did not cross the alpha boundary of significance, indicating insufficient statistical significance favoring the prolonged over the intermittent infusion group (Fig. 7). Moreover, the cumulative *Z*-curve also did not cross the TSA boundary, and the calculated diversity-adjusted information size (1850 patients) was not reached, which indicated an insufficient number of studies.

## Discussion

In this systematic review, we summarized the current evidence for a β-lactam antibiotics intravenous infusion strategy in sepsis or septic shock patients. Our study demonstrated that the prolonged infusion significantly improved compared to the intermittent infusion when the target plasma concentration and clinical cure were attained. Furthermore, the adverse event and occurrence of antibiotic-resistant bacteria did not increase in the prolonged infusion group. The hospital mortality, however, did not significantly differ between the groups.

β-Lactam antibiotics are time-dependent drugs, and their antibacterial activity is related to the duration of the maintenance of its concentration level exceeding the MIC. Patients who received β-lactams by continuous infusion were ten times more likely to exceed the target MIC than patients who received intermittent infusion [16]. Thus, our results could show a high clinical cure because of the high attainment of the target plasma concentration. Based on the pharmacokinetic/pharmacodynamics principle and our results, physicians should consider the prolonged infusion of β-lactam antibiotics strategy in treating sepsis or septic shock.

Clinical evidence supporting improved hospital mortality with prolonged β-lactams for sepsis or septic shock has been controversial [6, 8, 9, 29]. To solve this inconsistency, we added a subgroup analysis according to the year the studies were published. Interestingly, recent studies showed favorable results of prolonged infusion for hospital mortality whereas older studies did not. A possible reason for this result was the gradual increase in the reliance on pharmacokinetic/pharmacodynamics principles for the optimization of doses of antimicrobials. Another possibility was the improvement of study designs as recent studies were based on past studies. The results of the subgroup analysis indicated favorable effects of prolonged infusion in or after the year 2015. Moreover, we investigated using TSA to verify the strength of our results and detect reasons for conflicting results from previous studies [8, 9]. Finally, the analysis revealed an insufficient number of studies to reach a definitive conclusion for this topic. Further studies are needed and will provide clinicians with stronger confidence to adopt the prolonged infusion strategy for patients with sepsis or septic shock.

In 2019, a systematic review was performed, but the results were insufficient and could not differentiate between the continuous infusion and the traditional intermittent infusions of antibiotics [29]. However, the target population of a recent systematic review on an infectious disease (not sepsis) showed a high heterogeneity for the included studies. Nonetheless, our meta-analysis indicated low heterogeneity for the included studies, resulting in the inability of prolonged β-lactams in showing the superiority of the outcomes. Another previous systematic review showed improved hospital mortality in the prolonged infusion of β-lactam antibiotics [9]. This previous systematic study examined only antipseudomonal β-lactams, and some results of the sensitivity analysis could not show any differences in mortality, which correlated with our subgroup analysis results. Furthermore, the study did not take into account adverse events or resistant strains.

Our current systematic review examined the adverse events and the occurrence of antibiotic-resistant bacteria. Intermittent infusion resulted in a high number of adverse events because of the high peak of concentration in the intermittent infusion. However, no difference was observed, and this might be because β-lactams are generally considered to have a high safety window even when high doses are used [30]. Regarding the occurrence of antibiotic-resistant bacteria, very little data exist that describe the prevention of bacterial resistance caused by prolonged infusion. Theoretically, the bacterial occurrence of antibiotic-resistant bacteria should not increase because of prolonged infusion as a high clinical cure was shown in the prolonged infusion group. Our results therefore support the assertion that the prolonged infusion strategy could be safely performed without adverse events or an increase in antibiotic-resistant bacteria.

Caution, however, is required in our systematic review process. We defined continuous and extended infusion as “prolonged infusion” to avoid possible eligible studies. In vitro evidence demonstrated time periods where the free drug concentration exceeded the MIC for both extended and continuous infusions [6]. Several previous studies also used “prolonged infusion” in research settings [31, 32].

Our meta-analysis has several limitations. First, the outcomes may not apply to older patients as the mean age of enrolled patients was relatively young. For older patients, renal function could have deteriorated, resulting in a change of plasma concentration for antibiotics. Second, there were only a few RCTs included in our analysis for several secondary outcomes: attainment of target plasma concentration, adverse events, and the occurrence of antibiotic-resistant bacteria. More RCTs are needed in the future to support the results of our meta-analysis. Third, participants and healthcare staff were aware of the group assignments in some of the included RCTs, which could have resulted in performance bias. Therefore, we downgraded the certainty of the evidence in our results. Fourth, we defined intermittent infusion time as within 1 h because this cutoff value was used in previous studies [33, 34]. In contrast, we defined prolonged infusion time as over 1 h to include all possible studies. The cutoff time may affect the results although our study included only continuous infusion in the prolonged infusion group. Lastly, the subgroup analysis was performed retrospectively according to the publishing year (before and after 2015). The results might change if the analysis was conducted prospectively.

## Conclusions

The prolonged infusion of β-lactam antibiotics significantly improved as the target plasma concentration and a clinical cure were attained without increasing the number of adverse events or the occurrence of antibiotic-resistant bacteria. We could not show an improvement in hospital mortality in the prolonged infusion strategy despite recent studies of meta-analyses showing an improvement in hospital mortality in subgroup analysis. The TSA revealed an insufficient number of available studies to reach a definitive conclusion.

## Supplementary information

**Additional file 1.** Search strategies.

**Additional file 2.** Significant improvement for hospital mortality or clinical cure reported in studies published in or after 2015.

**Additional file 3.** Visual inspection of the funnel plot showing the absence of publication bias in hospital mortality.

**Additional file 4.** Summary of risk of bias graph for the primary outcome. (PPTX 67 kb)

## Data Availability

The data and material used for this meta-analysis are contained in our list of references.

## Abbreviations

ICU: Intensive care unit
MIC: Minimum inhibitory concentration
RCT: Randomized controlled trial
ICHUSHI: Igaku Chuo Zasshi
GRADE: Grading of Recommendations Assessment, Development, and Evaluation
RR: Risk ratio
CI: Confidence interval
