# Supplementary material for: Prolonged versus intermittent β-lactam antibiotics intravenous infusion strategy in sepsis or septic shock patients: a systematic review with meta-analysis and trial sequential analysis of randomized trials
Source: J Intensive Care. 2020 Oct 6;8:77. doi: 10.1186/s40560-020-00490-z (PMC7541232; doi:10.1186/s40560-020-00490-z)
Supplement: Supplementary file 1 — Additional file 1. Search strategies. [file 40560_2020_490_MOESM1_ESM.docx]

Additional file 1. Search Strategies in the systematic review

1. the MEDLINE database (via PubMed) search strategy（April 27, 2019）

| #1 | Sepsis [MeSH Terms]) OR Shock, Septic [MeSH Terms]) OR "Systemic inflammatory response syndrome" [MeSH Terms]) OR "Bacterial Infections" [MeSH Terms]) OR Sepsis [Title/Abstract]) OR Septic* [Title/Abstract]) OR Infection [MeSH Terms])) | 1409379 |
| --- | --- | --- |
| #2 | (beta-Lactams [MeSH Terms] OR carbapenem* [Title/Abstract] OR penicillin* [Title/Abstract] OR piperacillin [Title/Abstract]) OR cephalosporin* [Title/Abstract]) OR meropenem [Title/Abstract]) OR imipenem [Title/Abstract]) OR doripenem [Title/Abstract]) OR ticarcillin [Title/Abstract]) OR cefepime [Title/Abstract]) OR ceftazidime [Title/Abstract]) OR cefoperazone [Title/Abstract]) OR monobactam [Title/Abstract]) OR aztreonam [Title/Abstract])) OR Ertapenem [Title/Abstract]) OR Cefazolin [Title/Abstract]) OR "Clavulanic Acid*"[Title/Abstract]) OR Sulbactam [Title/Abstract]) OR Tazobactam [Title/Abstract])) OR beta-Lactam* [Title/Abstract]) | 181062 |
| #3 | (Drug Administration Schedule [MeSH Terms]) OR (extended [Title/Abstract]) OR prolonged [Title/Abstract]) OR continuous [Title/Abstract]) OR intermittent [Title/Abstract]) | 1019250 |
| #4 | Administration, Intravenous [MeSH Terms] OR infusion* [Title/Abstract]) OR administration* [Title/Abstract] | 1075028 |
| #5 | #3 AND #4 | 128269 |
| #6 | #1 AND #2 AND #5 | 1794 |
| #7 | ((“randomized controlled trial” [pt] OR “controlled clinical trial” [pt] OR randomized [tiab] OR placebo [tiab] OR “drug therapy” [sh] OR randomly [tiab] OR trial [tiab] OR groups [tiab])) | 4490408 |
| #8 | #6 AND #7 | 1494 |

2. the Cochrane Central Register of Controlled Trials search strategy

| #1 | MeSH descriptor: [Sepsis] explode all trees | 3998 |
| --- | --- | --- |
| #2 | MeSH descriptor: [Shock, Septic] explode all trees | 771 |
| #3 | MeSH descriptor: [Systemic Inflammatory Response Syndrome] explode all trees | 4328 |
| #4 | MeSH descriptor: [Bacterial Infections] explode all trees | 16094 |
| #5 | MeSH descriptor: [Infection] explode all trees | 22510 |
| #6 | MeSH descriptor: [Pneumonia] explode all trees | 3131 |
| #7 | (sepsis):ti,ab,kw | 10248 |
| #8 | (septic*):ti,ab,kw | 5100 |
| #9 | (bacteremia):ti,ab,kw | 2383 |
| #10 | ("critically ill"):ti,ab,kw | 5961 |
| #11 | (infect*):ti,ab,kw | 115779 |
| #12 | (pneumonia):ti,ab,kw | 13960 |
| #13 | #1 OR #2 OR #3 OR #4 OR #5 OR #6 OR #7 OR #8 OR #9 OR #10 OR #11 OR #12 | 140097 |
| #14 | MeSH descriptor: [beta-Lactams] explode all trees | 8954 |
| #15 | (carbapenem*):ti,ab,kw | 520 |
| #16 | (penicillin*):ti,ab,kw | 3531 |
| #17 | (piperacillin):ti,ab,kw | 917 |
| #18 | (cephalosporin*):ti,ab,kw | 2553 |
| #19 | (meropenem):ti,ab,kw | 593 |
| #20 | (imipenem):ti,ab,kw | 702 |
| #21 | (doripenem):ti,ab,kw | 96 |
| #22 | (ticarcillin):ti,ab,kw | 259 |
| #23 | (cefepime):ti,ab,kw | 339 |
| #24 | (ceftazidime):ti,ab,kw | 1045 |
| #25 | (cefoperazone):ti,ab,kw | 283 |
| #26 | (monobactam):ti,ab,kw | 25 |
| #27 | (aztreonam):ti,ab,kw | 386 |
| #28 | (ertapenem):ti,ab,kw | 193 |
| #29 | (cefazolin):ti,ab,kw | 1050 |
| #30 | (clavulanic acid*):ti,ab,kw | 1297 |
| #31 | (sulbactam):ti,ab,kw | 482 |
| #32 | (tazobactam):ti,ab,kw | 607 |
| #33 | (beta-lactam*):ti,ab,kw | 1182 |
| #34 | #14 OR #15 OR #16 OR #17 OR #18 OR #19 OR #20 OR #21 OR #22 OR #23 OR #24 #25 OR #26 OR #27 OR #28 OR #29 OR #30 OR #31 OR #32 OR #33 | 13798 |
| #35 | MeSH descriptor: [Drug Administration Schedule] explode all trees | 22968 |
| #36 | (extended):ti,ab,kw | 16633 |
| #37 | (prolonged):ti,ab,kw | 25010 |
| #38 | (continuous):ti,ab,kw | 46155 |
| #39 | (intermittent):ti,ab,kw | 12531 |
| #40 | #35 OR #36 OR #37 OR #38 OR #39 | 113693 |
| #41 | MeSH descriptor: [Administration, Intravenous] explode all trees | 17807 |
| #42 | (infusion*):ti,ab,kw | 61022 |
| #43 | (administration*):ti,ab,kw | 322182 |
| #44 | #41 OR #42 OR #43 | 350856 |
| #45 | #40 AND #44 | 56058 |
| #46 | #13 AND #34 AND #45 | 1362 |

3. the Igaku Chuo Zasshi (ICHUSHI; Japanese) database search strategy

| #1 | ((敗血症/TH or 敗血症/TA)) and (PT=会議録除く) | 22405 |
| --- | --- | --- |
| #2 | ((ショック-敗血症性/TH or 敗血症性ショック/TA)) and (PT=会議録除く) | 6145 |
| #3 | ((細菌感染症/TH or 細菌感染症/TA)) and (PT=会議録除く) | 115853 |
| #4 | ((全身性炎症反応症候群/TH or 全身性炎症反応症候群/TA)) and (PT=会議録除く) | 17730 |
| #5 | #1 or #2 or #3 or #4 | 132833 |
| #6 | ((Carbapenems/TH or carbapenems/TA or カルバペネム/TA or meropenem/TA or メロペネム/TA or imipenem/TA or イミペネム/TA or doripenem/TA or ドリペネム/TA or Ertapenem/TA or エルタペネム/TA)) and (PT=会議録除く) | 8823 |
| #7 | ((Penicillins/TH or penicillin/TA or ペニシリン/TA or piperacillin/TA or ピペラシリン/TA)) and (PT=会議録除く) | 17958 |
| #8 | ((Cephalosporins/TH or Cephalosporin/TA or セファロスポリン/TA or cefazolin/TA or セファゾリン/TA or ceftazidime/TA or セフタジジム/TA or cefepime/TA or セフェピム/TA or cefoperazone/TA or セフォペラゾン/TA)) and (PT=会議録除く) | 20924 |
| #9 | ((Beta-Lactams/TH or beta-lactams/TA or beta-lactam/TA)) and (PT=会議録除く) | 33334 |
| #10 | ((Beta-Lactamases/TH or beta-lactamase/TA or βラクタマーゼ/TA or sulbactam/TA or スルバクタム/TA or (clavulanic/TA and acid/TA) or クラブラン酸/TA or tazobactam/TA or タゾバクタム/TA)) and (PT=会議録除く) | 4614 |
| #11 | ((Monobactams/TH or monobactam/TA or モノバクタム/TA or aztreonam/TA or アズトレオナム/TA)) and (PT=会議録除く) | 1030 |
| #12 | #6 or #7 or #8 or #9 or #10 or #11 | 42645 |
| #13 | ((投薬計画/TH or 投薬計画/TA or 持続投与/TA or 長時間投与/TA or 投与時間延長/TA or 間欠投与/TA)) and (PT=会議録除く) | 36650 |
| #14 | ((静脈内投与/TH or 静脈内投与/TA)) and (PT=会議録除く) | 36165 |
| #15 | #13 AND #14 | 4870 |
| #16 | #5 AND #12 AND #15 | 134 |
| #17 | (ランダム化比較試験/TH or 準ランダム化比較試験/TH or ランダム化/AL or 無作為化/AL or 比較試験/AL or 臨床試験/AL or プラセボ/AL or 対照/AL or コントロール/AL or 臨床研究/AL or 治験/AL) and (PT=会議録除く) | 249263 |
| #18 | #16 AND #17 | 13 |
