# Supplementary material for: Prolonged versus intermittent β-lactam antibiotics intravenous infusion strategy in sepsis or septic shock patients: a systematic review with meta-analysis and trial sequential analysis of randomized trials
Source: J Intensive Care. 2020 Oct 6;8:77. doi: 10.1186/s40560-020-00490-z (PMC7541232; doi:10.1186/s40560-020-00490-z)
Supplement: Supplementary file 2 — Additional file 2. Significant improvement for hospital mortality or clinical cure reported in studies published in or after 2015. [file 40560_2020_490_MOESM2_ESM.pptx]

## Slide 1
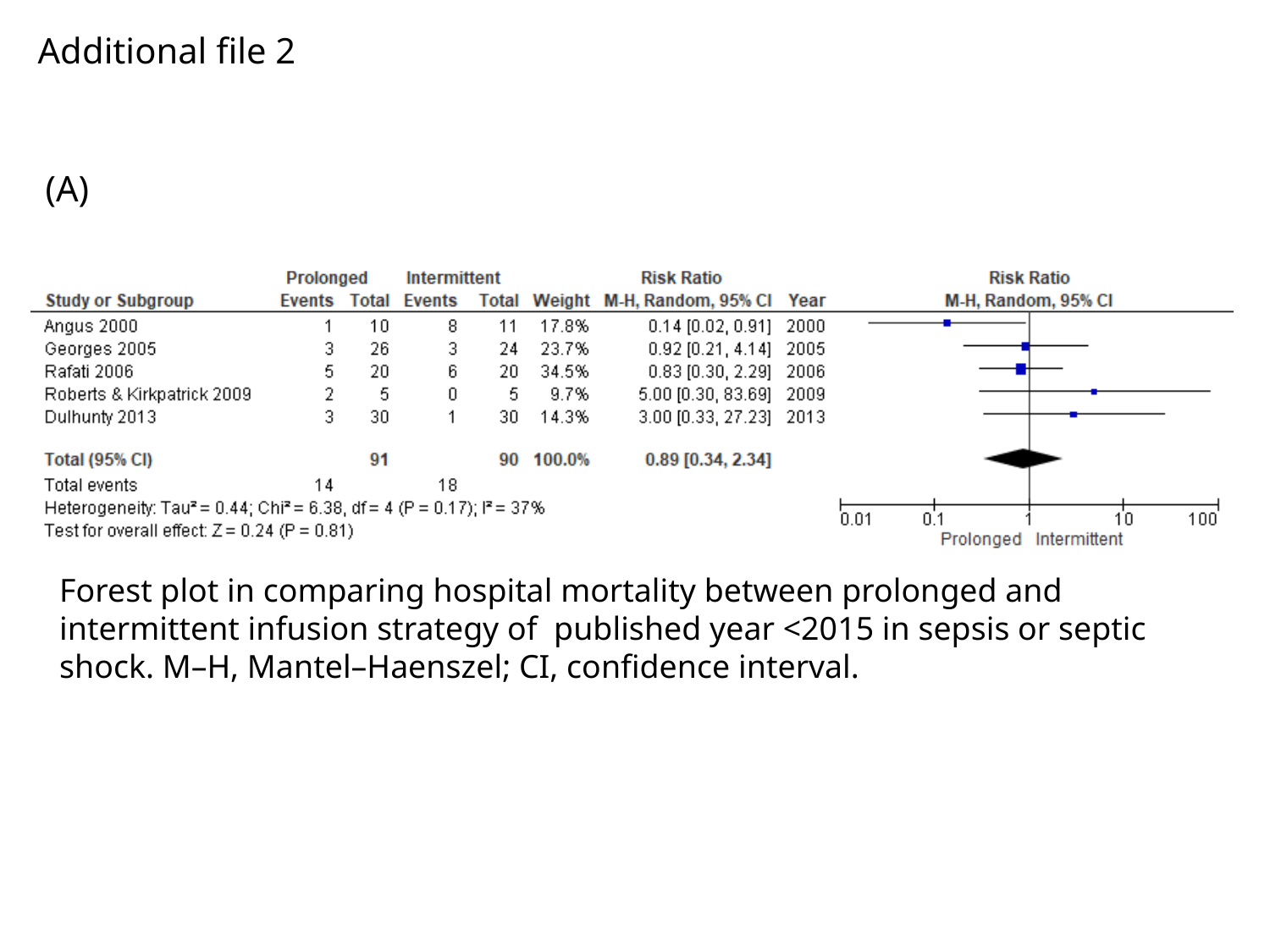

Additional file 2
(A)
Forest plot in comparing hospital mortality between prolonged and intermittent infusion strategy of published year <2015 in sepsis or septic shock. M–H, Mantel–Haenszel; CI, confidence interval.

## Slide 2
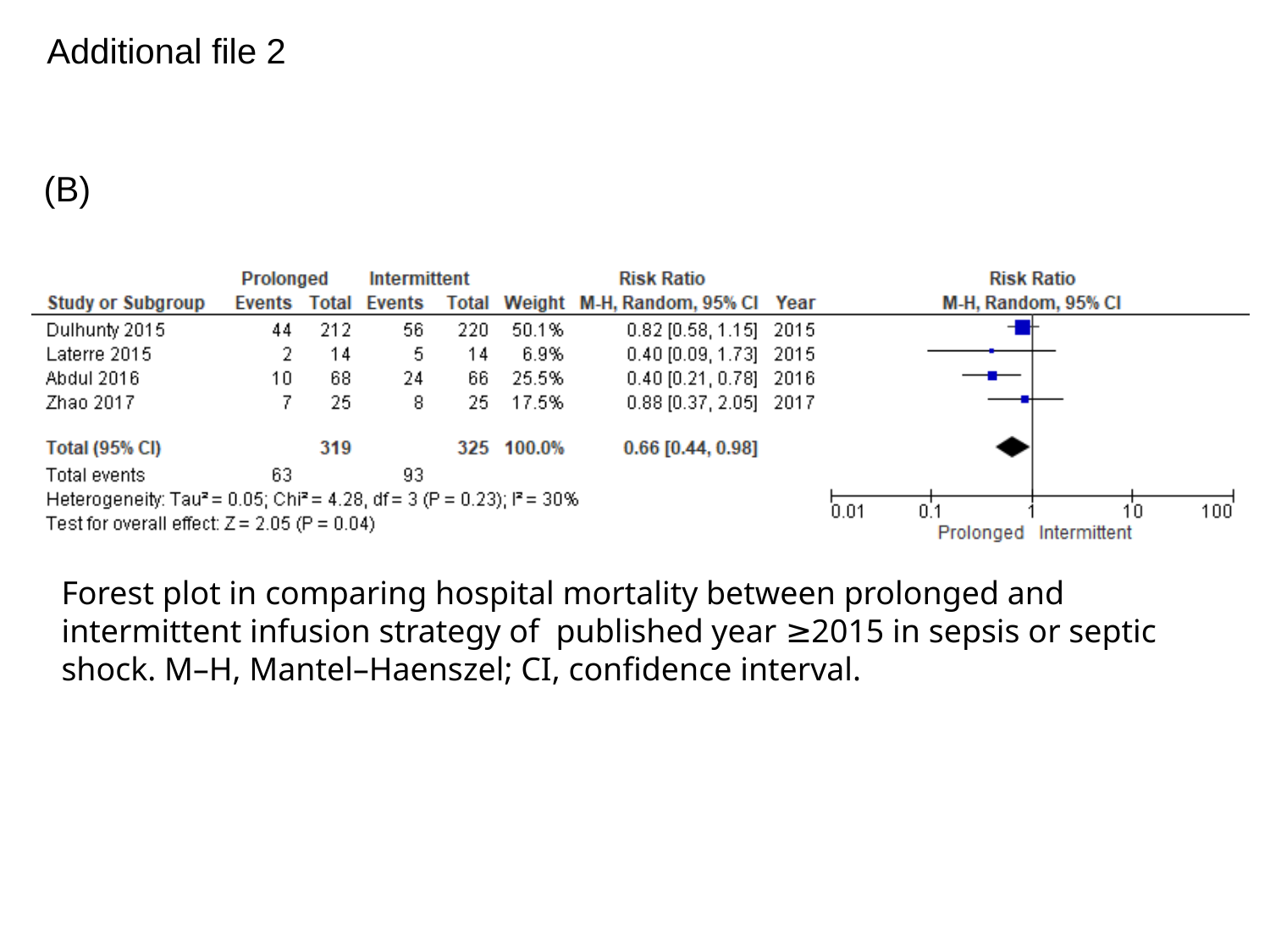

Additional file 2
(B)
Forest plot in comparing hospital mortality between prolonged and intermittent infusion strategy of published year ≥2015 in sepsis or septic shock. M–H, Mantel–Haenszel; CI, confidence interval.

## Slide 3
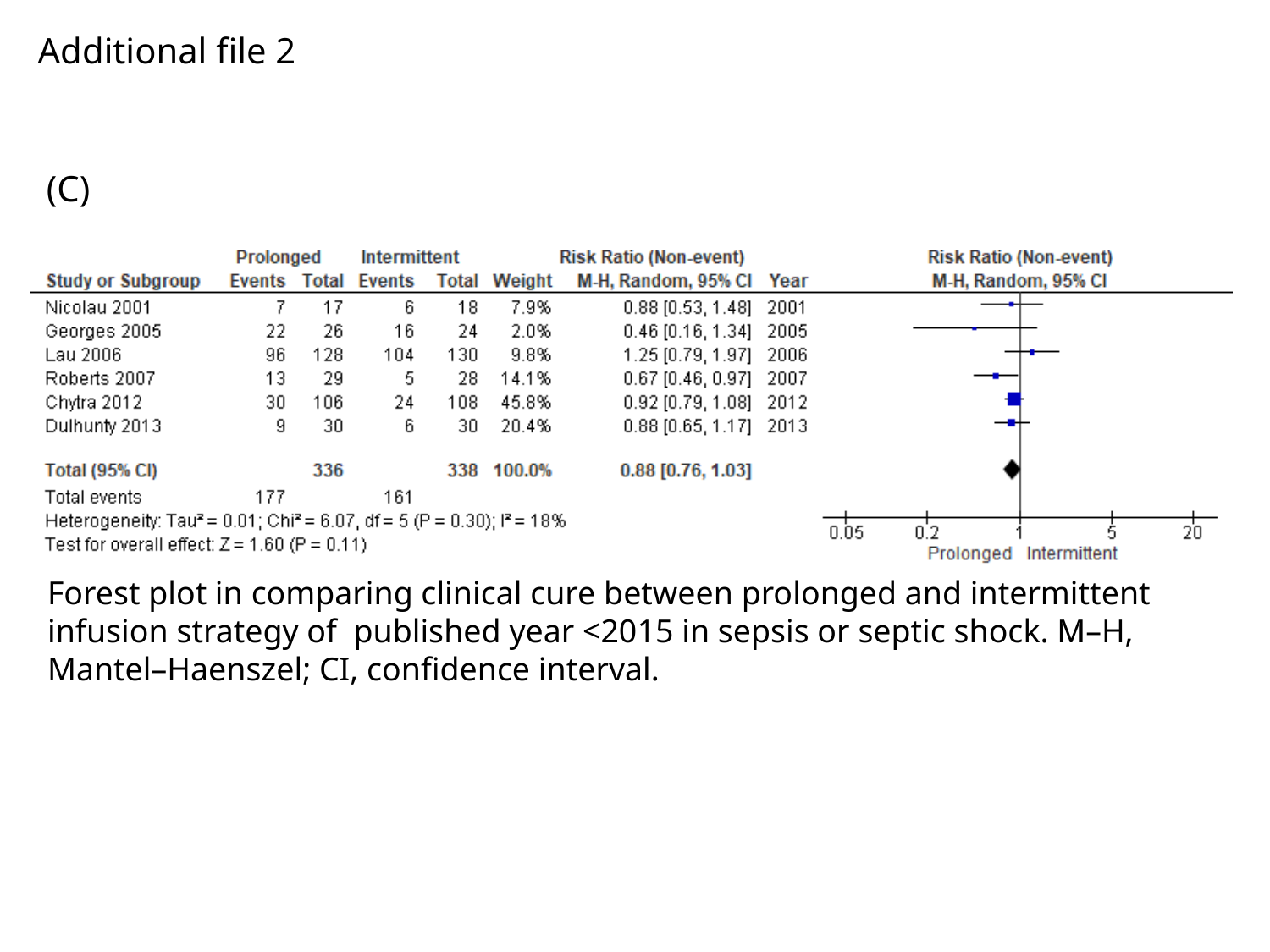

Additional file 2
(C)
Forest plot in comparing clinical cure between prolonged and intermittent infusion strategy of published year <2015 in sepsis or septic shock. M–H, Mantel–Haenszel; CI, confidence interval.

## Slide 4
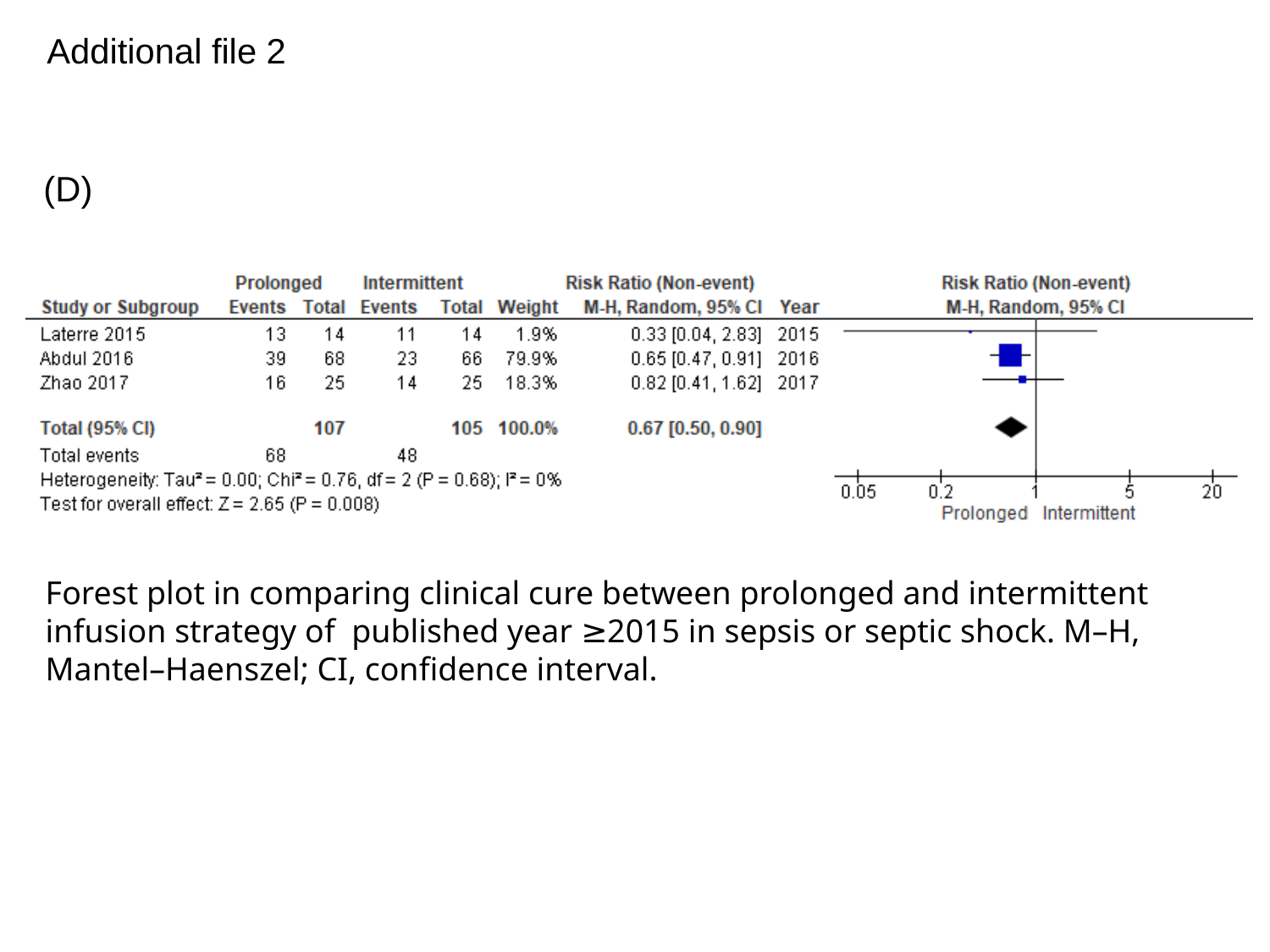

Additional file 2
(D)
Forest plot in comparing clinical cure between prolonged and intermittent infusion strategy of published year ≥2015 in sepsis or septic shock. M–H, Mantel–Haenszel; CI, confidence interval.
