# Supplementary material for: Prolonged versus intermittent β-lactam antibiotics intravenous infusion strategy in sepsis or septic shock patients: a systematic review with meta-analysis and trial sequential analysis of randomized trials
Source: J Intensive Care. 2020 Oct 6;8:77. doi: 10.1186/s40560-020-00490-z (PMC7541232; doi:10.1186/s40560-020-00490-z)
Supplement: Supplementary file 3 — Additional file 3. Visual inspection of the funnel plot showing the absence of publication bias in hospital mortality. [file 40560_2020_490_MOESM3_ESM.pptx]

## Slide 1
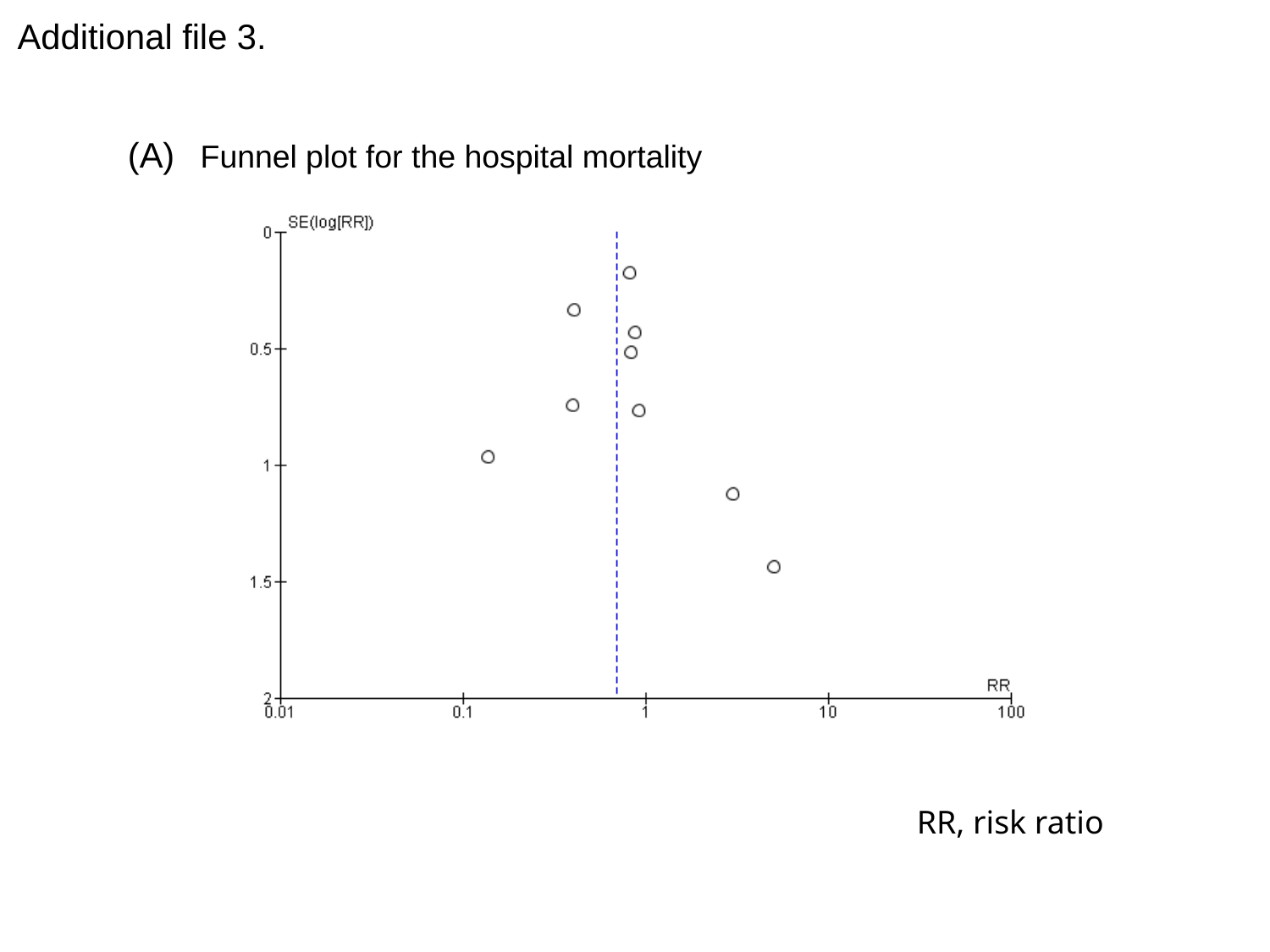

Additional file 3.
(A)
Funnel plot for the hospital mortality
RR, risk ratio

## Slide 2
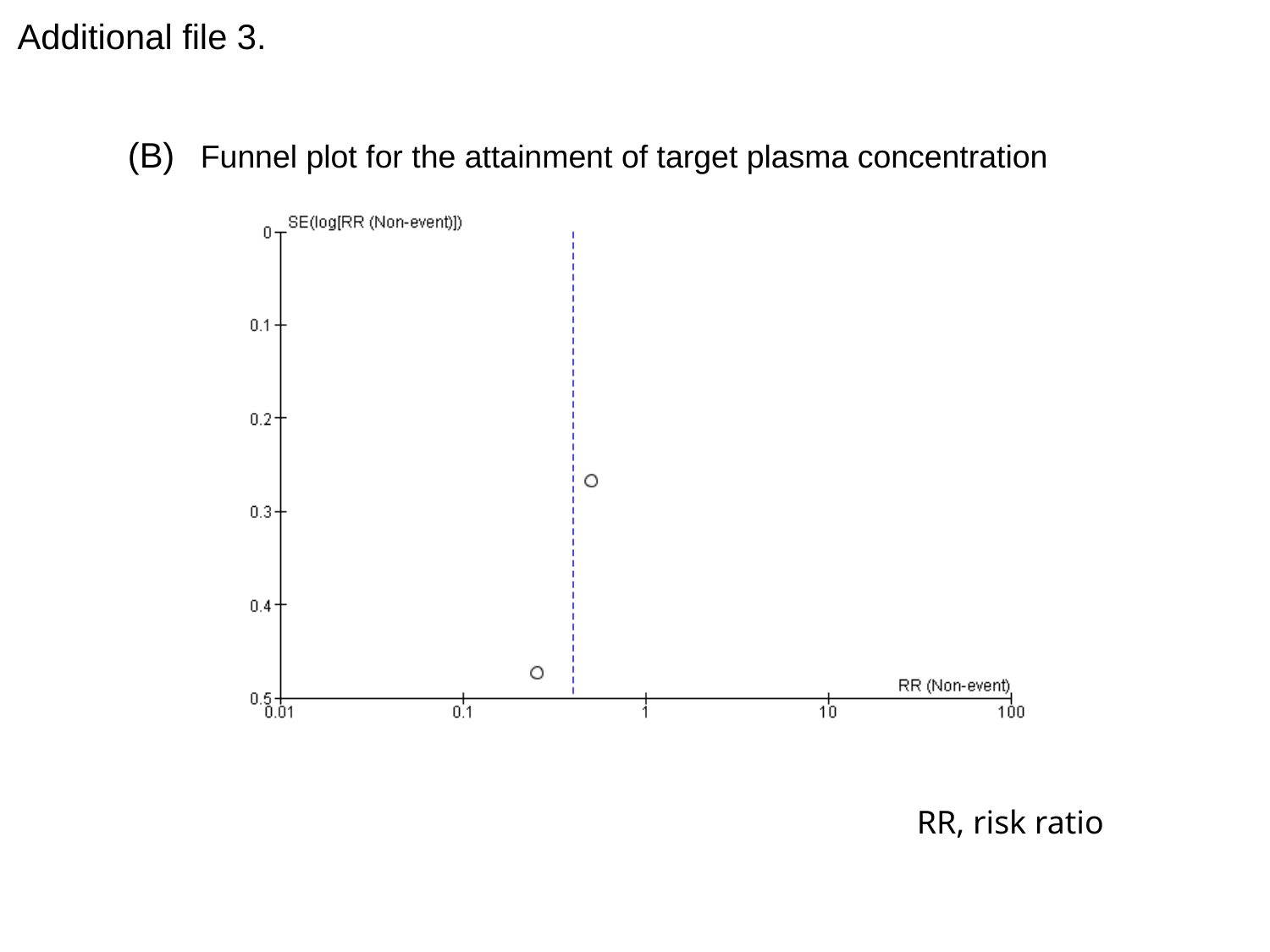

Additional file 3.
(B)
Funnel plot for the attainment of target plasma concentration
RR, risk ratio

## Slide 3
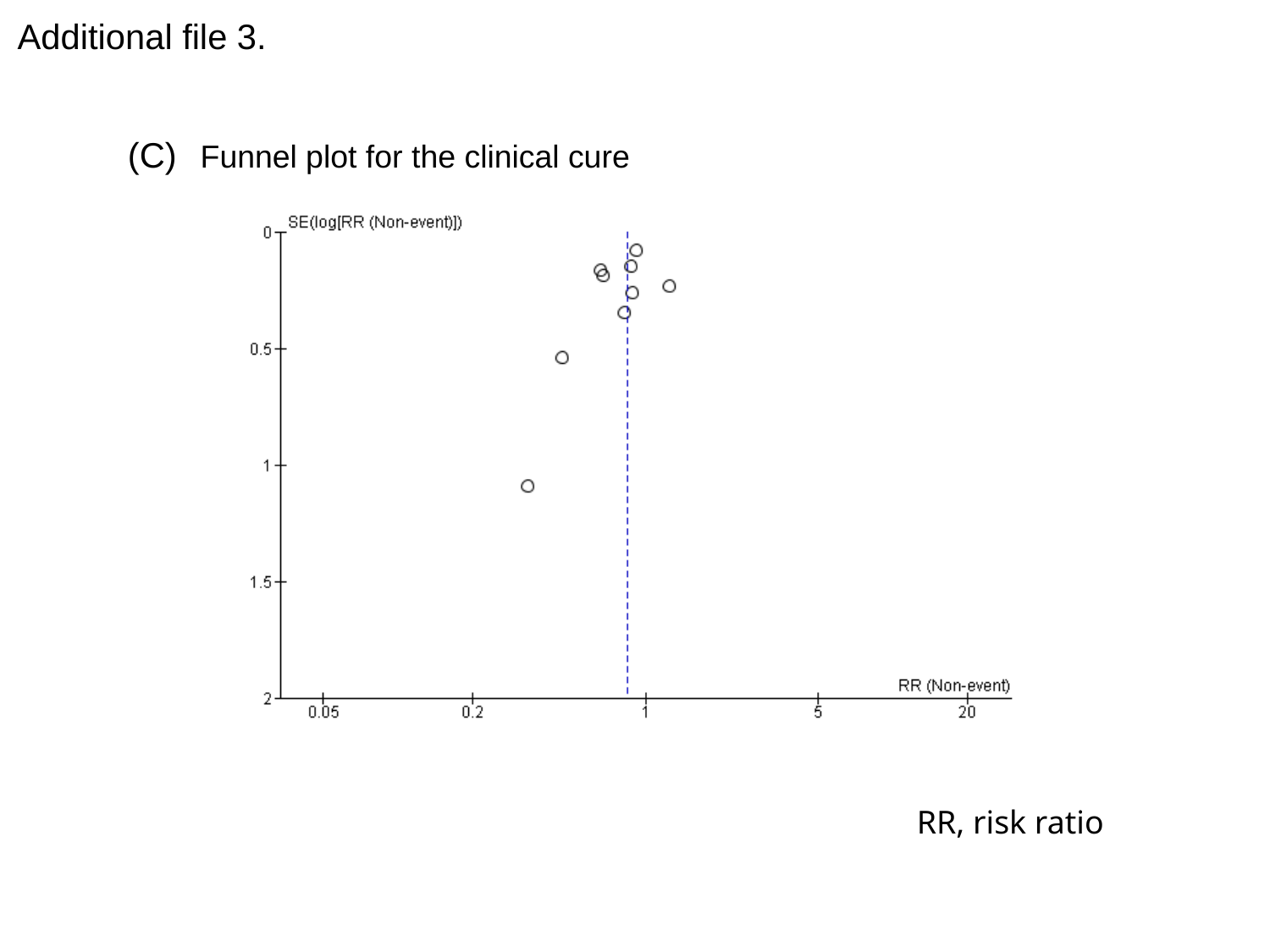

Additional file 3.
(C)
Funnel plot for the clinical cure
RR, risk ratio

## Slide 4
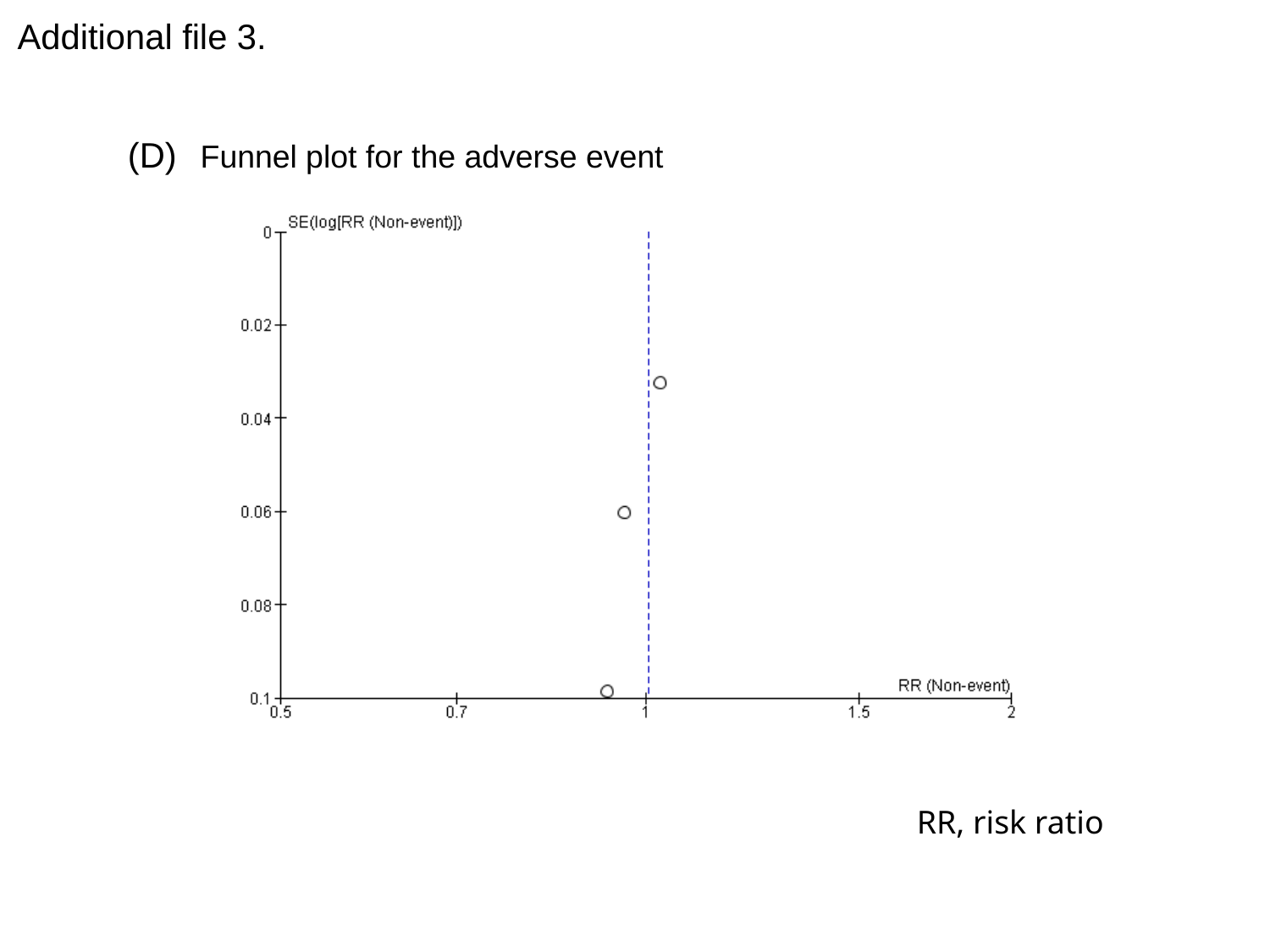

Additional file 3.
(D)
Funnel plot for the adverse event
RR, risk ratio

## Slide 5
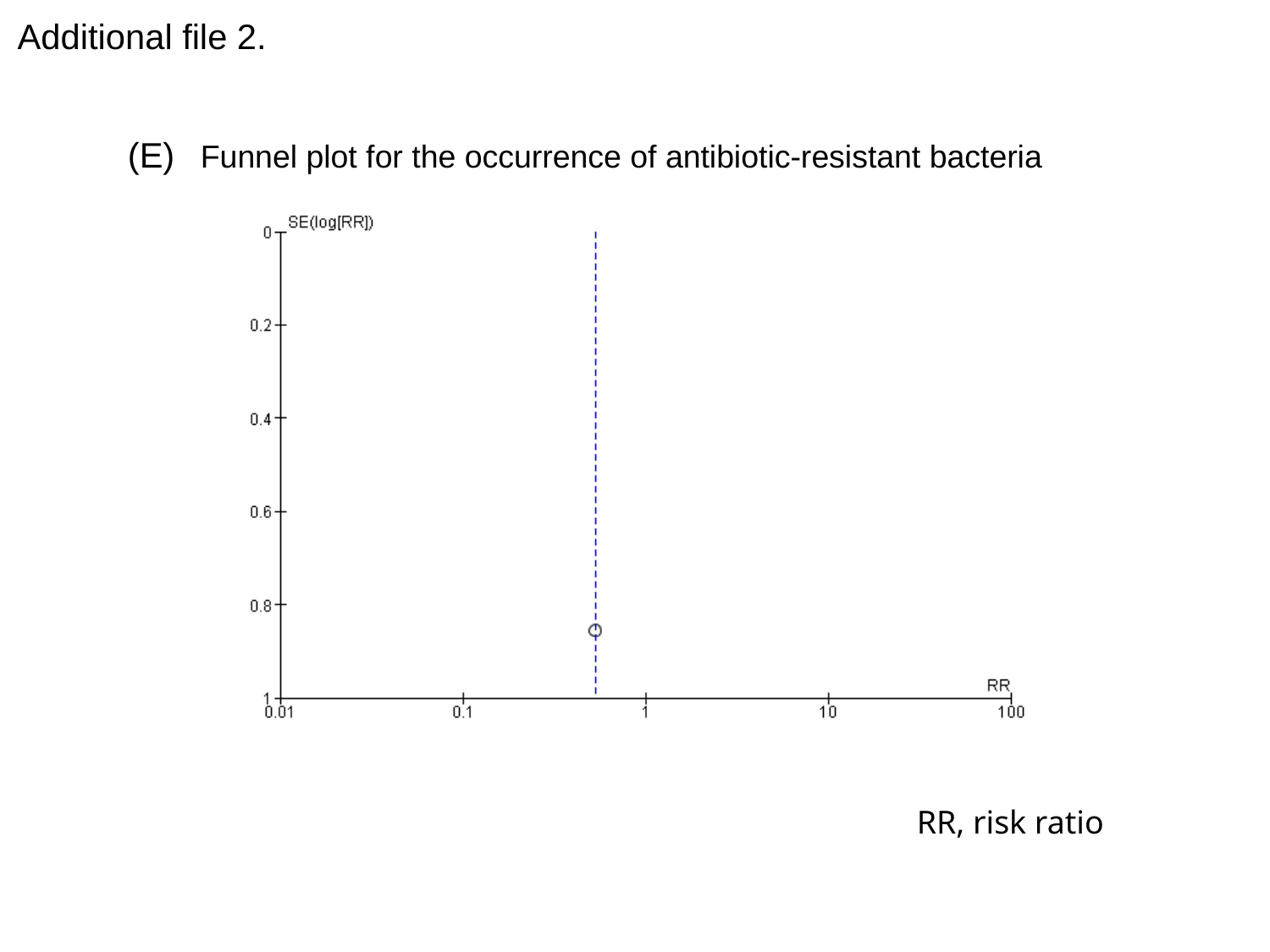

Additional file 2.
(E)
Funnel plot for the occurrence of antibiotic-resistant bacteria
RR, risk ratio
