# Supplementary figures and images for: Prolonged versus intermittent β-lactam antibiotics intravenous infusion strategy in sepsis or septic shock patients: a systematic review with meta-analysis and trial sequential analysis of randomized trials
Source: J Intensive Care. 2020 Oct 6;8:77. doi: 10.1186/s40560-020-00490-z (PMC7541232; doi:10.1186/s40560-020-00490-z)

## Slide 1
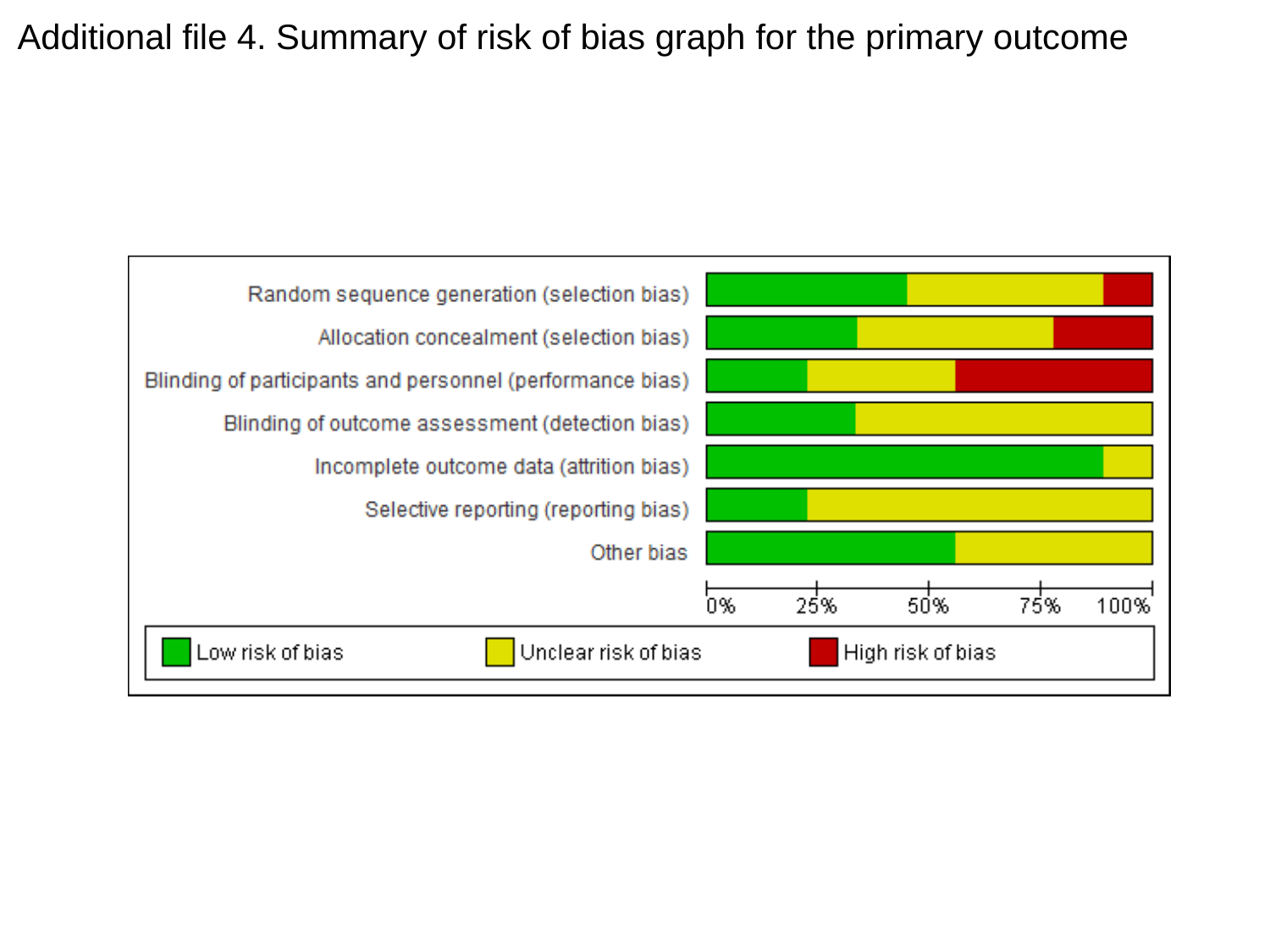

Additional file 4. Summary of risk of bias graph for the primary outcome

Supplement: Supplementary file 4 — Additional file 4. Summary of risk of bias graph for the primary outcome. (PPTX 67 kb) [file 40560_2020_490_MOESM4_ESM.pptx]
